# Specificities and Synergistic Actions of Novel PL8 and PL7 Alginate Lyases from the Marine Fungus *Paradendryphiella salina*

**DOI:** 10.3390/jof7020080

**Published:** 2021-01-25

**Authors:** Bo Pilgaard, Marlene Vuillemin, Jesper Holck, Casper Wilkens, Anne S. Meyer

**Affiliations:** Section for Protein Chemistry and Enzyme Technology, Department of Biotechnology and Biomedicine, Technical University of Denmark, Søltofts Plads, Building 221, DK-2800 Kgs. Lyngby, Denmark; bpil@dtu.dk (B.P.); mavu@dtu.dk (M.V.); jesho@dtu.dk (J.H.); cwil@dtu.dk (C.W.)

**Keywords:** alginate lyase, polysaccharide lyase, marine fungi, enzyme characterization, alginate, polymannuronic acid, polyguluronic acid, exo-acting

## Abstract

Alginate is an anionic polysaccharide abundantly present in the cell walls of brown macroalgae. The enzymatic depolymerization is performed solely by alginate lyases (EC 4.2.2.x), categorized as polysaccharide lyases (PLs) belonging to 12 different PL families. Until now, the vast majority of the alginate lyases have been found in bacteria. We report here the first extensive characterization of four alginate lyases from a marine fungus, the ascomycete *Paradendryphiella salina*, a known saprophyte of seaweeds. We have identified four polysaccharide lyase encoding genes bioinformatically in *P. salina*, one PL8 (PsMan8A), and three PL7 alginate lyases (PsAlg7A, -B, and -C). PsMan8A was demonstrated to exert exo-action on polymannuronic acid, and no action on alginate, indicating that this enzyme is most likely an exo-acting polymannuronic acid specific lyase. This enzyme is the first alginate lyase assigned to PL8 and polymannuronic acid thus represents a new substrate specificity in this family. The PL7 lyases (PsAlg7A, -B, and -C) were found to be endo-acting alginate lyases with different activity optima, substrate affinities, and product profiles. PsAlg7A and PsMan8A showed a clear synergistic action for the complete depolymerization of polyM at pH 5. PsAlg7A depolymerized polyM to mainly DP5 and DP3 oligomers and PsMan8A to dimers and monosaccharides. PsAlg7B and PsAlg7C showed substrate affinities towards both polyM and polyG at pH 8, depolymerizing both substrates to DP9-DP2 oligomers. The findings elucidate how *P. salina* accomplishes alginate depolymerization and provide insight into an efficient synergistic cooperation that may provide a new foundation for enzyme selection for alginate degradation in seaweed bioprocessing.

## 1. Introduction

Marine fungi are widespread in various marine environments all over the Globe ranging from deep-sea to surface waters [1]. These fungi appear to play numerous ecological roles as saprobes and pathogens of algae and plankton in addition to being symbionts of coral reef animals [2]. There is currently only 1100 species described as marine fungi although it is hypothesized, based on simple extrapolation, that there exists more than 10,000 species [3]. These numbers are dwarfed by the 60,000 described and the estimated 1.5 million terrestrial fungal species [4], and emphasize the lacking knowledge of marine fungi. One of the interesting questions surrounding marine fungi pertains to their origin; more specifically, whether they have evolved in the marine environment or simply adapted to it. While both scenarios are likely, depending on the taxonomy, studies suggest that marine asco- and basidiomycete species may predominantly have transitioned from terrestrial to marine life [2,5]. One such ascomycete suspected to have transitioned in this way is the hyphomycete *Paradendryphiella salina*, which consistently has been associated with saprotrophy of brown algae [6,7,8,9].

*P. salina* belongs to the class of Dothideomycetes and appears melanized, which suggests it belongs to the group of fungi called black fungi or black yeasts. These fungi are often found in the marine environment and are known for their high stress tolerance to abiotic factors such as high UV-radiation, low nutrient availability, and high salinity [10].

Alginate is a polysaccharide found abundantly in the cell wall matrix of brown algae. It can constitute as much as 40% of the cell wall and represents the first line of defense towards invading microorganisms such as fungi [11]. Alginate is a linear heteropolymer consisting of the two uronic acids; β-d-mannuronic acid (M) and its C5 epimer α-l-guluronic acid (G), these are linked by 1,4-*O*-linked glycosidic bonds. The two monomers are primarily found in sequences of M (M-blocks) or G (G-blocks), but also in interchangeable MG-blocks. The total amount of alginate and M- and G-content can vary across species of brown seaweed and across seasons [12]. Estimated annual sales volumes of alginate from brown algae was 30,000 metric tons in 2014 data [13]. Alginate has wide applications as a viscosity enhancing hydrocolloid, gelling agent, and as a food and cosmetics additive. In medicine it is for example used in wound dressing, cell immobilization, and drug delivery systems [14]. An increasing number of promising pharmaceutical applications of alginate oligosaccharides have arisen due to the oligosaccharides retaining most of the physical and chemical properties of alginate, but without the gel-forming quality. The oligosaccharides show promising results in e.g., treatment of multidrug resistant bacterial and fungal infections where very few alternatives exist [15]. There is therefore increasing attention towards discovering and characterizing alginate lyases that can depolymerize alginate in a specific and controlled manner, for production of tailored oligomers to specific applications.

Based on their amino acid sequence, alginate lyases are presently classified in 12 different polysaccharide lyase (PL) families in the Carbohydrate-Active-EnZYme-database (CAZy) (www.cazy.org) [16]. Alginate lyases catalyze alginate depolymerization by β-elimination, producing Δ4,5 unsaturated bonds at the nonreducing end of one of the cleavage products [16]. Almost all characterized alginate lyases are of bacterial origin, but a few originating from algae, animals, and vira have been identified [17,18,19,20]. The majority of the known alginate lyases are endo-acting and some display multiple specificities towards M- and G-blocks and to a lesser extent MG-blocks [21]. The specific reactions are classified into endo-acting polyM lyases (EC 4.2.2.3), endo-acting polyG lyases (EC 4.2.2.11), exo-acting oligo-alginate lyases (EC 4.2.2.26), and MG-lyases (EC 4.2.2.-).

In a previous study we genome sequenced *P. salina* CBS112865 and analyzed the genome and its secreted proteome when *P. salina* was growing on different species of brown algae. Amongst other enzymes we identified four lyases, which we hypothesized to be essential for the ability of *P. salina* to penetrate the brown algae cell walls and allow it to grow on the brown macroalgae. Three lyases from the PL7 family, that the phylogenetic analysis suggested to be alginate lyases, and one lyase from the PL8 family with unknown substrate specificity were tentatively identified via the genomic analysis. Two of the four lyases, specifically PsAlg7A and PsMan8A, were highly secreted by the fungus after 14 days of growth on different brown macroalgae species [22]. The objective of the present study was to obtain an understanding of *P. salina’s* ability depolymerize alginate by performing an in-depth characterization of these four enzymes with respect to substrate specificities, reaction optima, enzyme kinetics, product formation, and explore any possible synergies between the endo-exo functions of PsAlg7A and PsMan8A.

## 2. Materials and Methods

### 2.1. Substrates

Sodium alginate, Chondroitin AC, Chondroitin B, and Hyaluronic acid were purchased from Sigma (Sigma Aldrich, Saint-Louis, MO, USA). Polymannuronic acid (85% purity, >5000 Da) (polyM) and polyguluronic acid (80% purity) (polyG) were purchased from Carbosynth (Carbosynth, Compton, UK). PolyMG (DP30) was kindly donated by the Finn Aachmann lab (Trondheim, Norway).

### 2.2. Sequence Analysis

Putative catalytic domains of CAZymes were predicted by HMMER v. 3.3.1 [23] using the most recent (07-30-2020) Hidden Markov Models (HMMs) from the dbCAN server (http://bcb.unl.edu/dbCAN2) [24] by applying the default E-value (<1 × 10^−3^) and coverage (>0.3) cutoffs in the hmmscan-parser script from dbCAN. Additionally, the Interproscan server (https://www.ebi.ac.uk/interpro/) [25] was used as a tool for secondary conformational domain prediction along with prediction of proteins other than CAZymes. Signal peptide predictions were performed using the Phobius webserver (https://phobius.sbc.su.se/) [26]. *N*-glycosylation sites were predicted using the NetNGlyc 1.0 Server (www.cbs.dtu.dk/services/NetNGlyc) [27].

### 2.3. Phylogenic Analysis of PL8 Sequences

All PL8 protein sequences listed in CAZy including that of PsMan8A (VYG66350.1 (deposited by us)) were downloaded from GenBank (www.ncbi.nlm.nih.gov/) [28]. The phylogenic analysis was performed by aligning the dbCAN predicted catalytic domain sequences with Mafft [29], which were manually inspected in CLC main workbench (8). Maximum likelihood analyses were performed with RaxML blackbox [30] using LeGascuel substitution matrix [31] and otherwise default parameters at the CIPRESS server (www.phylo.org/) [32]. RaxML stopped the rapid bootstrap search after 360 replicates with the MRE-based Bootstopping criterion.

### 2.4. Genes, Cloning, Expression, and Purification of PsAlg7A, -B, -C, and PsMan8A Alginate Lyases

Cloning, expression, and purification of PsAlg7A (GenBank accession VFY81779.1), PsAlg7B (GenBank accession CAD6594632.1) and PsAlg7C (GenBank accession CAD6594633.1) were performed as previously described [22]. The open reading frame encoding PsMan8A was identified in the *P. salina* genome (GenBank accession GCA_900634815.1) as described previously [22]. The mature gene (GenBank accession VYG66350.1) excluding a 23 amino acids long predicted signal peptide, but including a C-terminal 6×His-tag, was codon optimized for *Pichia pastoris* expression, synthesized by GenScript (Piscataway, NJ, USA), and cloned into the pPink-HC vector (Invitrogen, Carlsbad, CA, USA). The resulting construct was transformed into *Escherichia coli* strain EPI400 and selected on LB agar plates supplemented with 100 μg mL^−^^1^ ampicillin. The pPink-HC-PsMan8A construct was linearized using PmeI (New England BioLabs, Ipswich, MA, USA) and transformed into *P. pastoris* PichiaPink strain 4 as previously described [33]. White transformants were selected on PAD plates (Invitrogen). Expression and purification of all recombinant proteins were performed as previously described [22]. The theoretical molar extinction coefficient and sizes of the recombinant enzymes were calculated using ProtParam (http://web.expasy.org/protparam) [34] (Supplementary Material, Table S1).

Protein concentrations were determined by A_280_ using the theoretically obtained molar extinction coefficients (Table S1). Deglycosylation of PsAlg7B and PsMan8A was performed by adding EndoH (New England Biolabs, Ipswich, MA, USA) to the purified glycoproteins and incubating at 37 °C for 1 h. The purity of the recombinant proteins and the effect of the EndoH treatment were checked on SDS-PAGE gels (Figure S1).

### 2.5. Standard Assay Conditions

Alginate lyase activity was determined by monitoring the formation of the 4,5-unsaturated bonds at the nonreducing end by following the absorbance at A_235_ at regular time intervals for 10 min spectrophotometrically. Reactions were carried out at optimum temperatures, 35 °C for PsAlg7A, 40 °C for PsAlg7B and PsAlg7C, and 25 °C for PsMan8A, using 1.5 g·L^−^^1^ of substrate in 20 mM UB4 buffer [35] at optimum pH, pH 5 for PsAlg7A and PsMan8A and pH 8 for PsAlg7B and PsAlg7C, and 150 mM NaCl for PsAlg7A and PsMan8A and 250 mM NaCl for PsAlg7B and PsAlg7C in a 96-well quartz plate (Corning, New York, NY, USA). All measurements were performed in triplicates. One unit (U) was defined as the amount of the enzyme required to increase by 0.1 absorbance units at 235 nm (A_235_) per minute.

### 2.6. Biochemical Characterization

The substrate specificity was assessed for each enzyme under standard assay conditions and using 1.5 g·L^−^^1^ of substrate (alginate, polyM, polyG, polyMG, chondroitin AC, chondroitin B, or hyaluronic acid,) in 20 mM UB4 buffer at three different pH values (pH 4, 6, and 8). Activities were quantified by monitoring the absorbance at A_235_, as described above. The pH optimum was determined for each enzyme under standard assay conditions using 1.5 g·L^−^^1^ of substrate in 20 mM UB4 buffer ranging from pH 2 to 8 for PsAlg7A and PsMan8A and in 20 mM MIB (malonic acid, imidazole, boric acid) buffer ranging from pH 4 to 10 for PsAlg7B and PsAlg7C. The optimum temperature was determined for each enzyme under standard assay conditions within a temperature range of 10–50 °C PsAlg7A and PsMan8A and 25–50 °C for PsAlg7B and -C.

The effect of NaCl on enzyme activity was investigated under standard assay conditions with varying NaCl concentrations (15–500 mM). The effects of divalent ions and EDTA on the activity of the enzyme were analyzed by incubating the purified enzyme at 4 °C for 24 h with CaCl_2_, ZnCl_2_, MgCl_2_, NiCl_2_, and MnCl_2_ at a final concentration of 2 mM and measuring the activity under standard conditions. Prior to the incubations, the enzymes were incubated with 2 mM EDTA for 10 min, and then dialyzed twice against the elution buffer.

### 2.7. Kinetics of PsAlg7A, PsAlg7B, PsAlg7C, and PsMan8A

For each enzyme, initial velocities were quantified on sodium alginate, polyM, and polyG (0.25 to 15 g·L^−^^1^) under standard assay and optimum conditions. The average initial velocities quantified in milli-absorbance units (mAU) at A_235_ per second were converted to mM of product formed via measuring the amount of 4,5-unsaturated bonds formed per second using the extinction coefficient of 6150 M^−^^1^ cm^−^^1^ [36,37]. Kinetic parameters were determined for each enzyme by plotting initial velocities, quantified as above, against initial substrate concentration. *K*_m_ and *k*_cat_ were obtained by fitting the Michaelis–Menten equation *v*_o_ = *V*_max_**/**(1 + (*K*_m_/[S_0_]) using Origin (OriginLab Corporation, Northampton, MA, USA) where *v*_o_ is the initial velocity, [S_0_] the initial substrate concentration, *V*_max_ the maximum rate, and *K*_m_ the Michaelis constant.

### 2.8. Synergy of PsAlg7A and PsMan8A

Synergy was investigated by combining loadings of PsAlg7A and PsMan8A to a total of 0.2 μM enzyme in the reactions. The enzymes were incubated with 1.5 g·L^−^^1^ alginate or polyM, 150 mM NaCl and 2 mM ZnCl_2_, in UB4 buffer pH 5 at 30 °C. Activity was measured as described above under standard assay conditions. Degree of Synergy (DS) were defined as the ratio between the theoretical activity without synergy and the Specific Activity (SA) [38].

### 2.9. Action Pattern and Degradation Product Analysis by LC-MS

Duplicate reactions were prepared under standard assay conditions. Samples were taken at different reaction times (10, 20, 60, and 120 min). The reactions were stopped by addition of acetonitrile (Sigma) to a final concentration of 50%. Prior to LC/MS analysis each sample was spun down for 10 min at 10,000× *g*. Identification and relative quantification of 4,5-unsaturated alginate oligosaccharides was performed by liquid chromatography electrospray ionization mass spectrometry (LC-ESI-MS) on an Amazon SL iontrap (Bruker Daltonics, Bremen Germany) coupled to an UltiMate 3000 UHPLC equipped with an Ultimate RS diode array detector (Dionex, Sunnyvale, CA, USA). A 5 µL sample in 50% acetonitrile was injected on a GlycanPac AXH-1 column (150 mm × 2.1 mm, Thermo Fisher Scientific. Waltham, MA, USA). The chromatography was performed at 0.4 mL/min at 30 °C on a three-eluent system composed of eluent A (water), eluent B (100 mM ammonium formate pH 5), and C (acetonitrile). Eluent A was kept at 19% at all time. The elution profile was as follows (time indicated in min): 0–35, linear gradient to 1–19% B; 35–40, linear gradient to 1% B; 40–50, isocratic 1% B. The electrospray was operated in negative mode with UltraScan mode and a scan range from 100–2000 *m/z*, smart parameter setting of 500 *m/z*, capillary voltage at 4.5 kV, end plate off-set 0.5 kV, nebulizer pressure at 3.0 bar, dry gas flow at 12.0 L/min, and dry gas temperature at 280 °C. Relative quantification of compound intensities was performed in Compass QuantAnalysis 2.2 (Bruker Daltonics, Bremen, Germany). Compound intensities were deducted the substrate control reactions without enzymes.

## 3. Results

### 3.1. Genetic Environment

**Figure 1:**
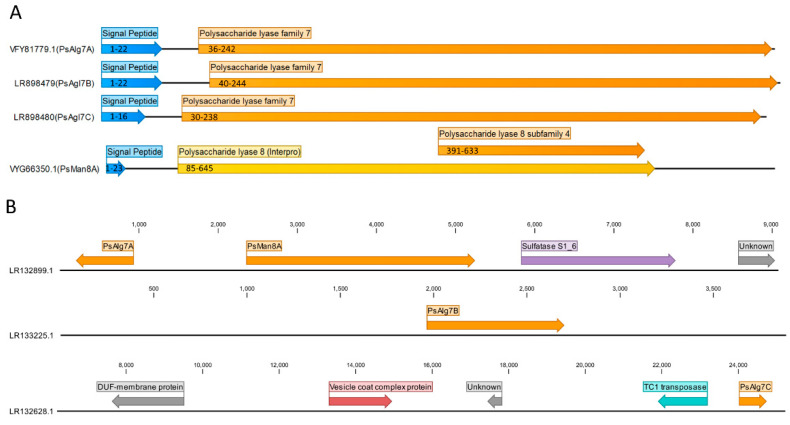
(**A**) HMM-based domain architecture of the four alginate lyase protein sequences. Signal peptides were predicted by Phobius (blue) and the catalytic domains by HMMer using dbCAN models (orange) and the Interproserver (yellow). The numbers indicate the location of the predicted domain on the full length sequence. (**B**) Contig-based genetic environment of the lyases. The contig accession numbers from GenBank are displayed in front of each sequence. The predicted genes, annotation, and transcription direction are represented by the colored arrows. The number-scale on each contig represents the nucleotide count.

The four genes were located on three different contigs in the *P. salina* genome. PsAlg7A and PsMan8A were located on the same contig (9079 nucleotides) next to each other in two different transcriptional directions. Downstream of them were a predicted sulfatase S1_6 gene and a gene of unknown function (Figure 1B). PsAlg7B was located on a small contig (3889 nucleotides) with no other predicted genes. PsALg7C was located on the longest of three contigs (25,230 nucleotides), but with only four other predicted genes of little or no relevance to alginate depolymerization (Figure 1B).

### 3.2. Sequence Analysis

The gene prediction and subsequent annotation of the *P. salina* genome revealed three genes without introns predicted to belong to the PL7 family. The corresponding proteins, PsALg7A, PsALg7B, and PsALg7C consisted of 243, 245, and 240 amino acids, respectively. The calculated molar weight of each of the proteins were approximately 25 kDa. The results from the Interproscan server were in agreement with the dbCAN predictions. All three harbored eukaryotic signal peptides of 19–22 amino acids long and a PL7 catalytic domain as predicted by Phobius and dbCAN, respectively (Figure 1A). The fourth gene, which sparked our attention due to it being located next to the PsAlg7A, contained several introns and the derived 786 amino acid long protein sequence (85 kDA) was predicted by the dbCAN HMM model to contain a PL8 lyase subfamily 4 catalytic domain. The sequence also harbored a 23 amino acids long eukaryotic signal peptide. The HMM model predicted the catalytic domain from amino acid position 391–633 with loose parameters (E-value < 1 × 10^−5^, coverage > 0.3). The Interproscan server predicted a longer PL8 lyase catalytic domain (IPR038970) from amino acid position 85–645 (Figure 1A).

### 3.3. Blastp Analysis

A BLASTp analysis revealed the top hits of the results of the three PL7 lyases to share the highest identity with each other at approximately 50% and the remaining closest hits belonged to putative PL7 proteins amongst various marine bacteria with the highest identity of 33%. The result of PsMan8A returned only terrestrial fungal sequences of putative PL8 proteins within the first 100 hits. The closest hit was a putative Chondroitinase-AC from *Tolypocladium ophioglossoides* (KND87076.1) sharing a 63% sequence identity with PsMan8A, this sequence was however not found in CAZy. The closest sequence in the PL8 family in CAZy was similarly a putative Chondroitinase-AC from Metarhizium brunneum (QLI65685.1), which share 59% identity with PsMan8A.

### 3.4. Phylogenetic Analysis

**Figure 2:**
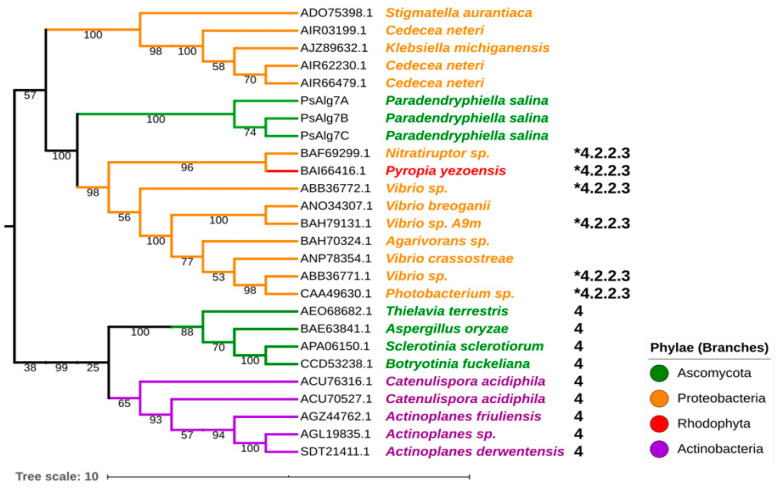
Maximum likelihood tree of selected PL7 family sequences from the CAZy database. The * indicate EC number of characterized members (4.2.2.3 = polyM-specific alginate lyase). The single numbers indicate subfamily classification from CAZy. The branch colors indicate phylae. Branch numbers indicate bootstrap values. The tree scale bar indicates substitution changes per site. This tree is a pruned and updated version of the tree presented in a previous study [22].

The phylogenetic relationship of the three PsPL7s has been described in an earlier study [22]. In brief, the three PL7 alginate lyases from *P. salina* clustered together in a major clade dominated by marine proteobacteria [22]. The only other sequence of eukaryotic origin in this clade belongs to the red seaweed *Pyropia yezoensis*. The data infer that the three *P. salina* PL7 sequences constitute their own subclade with no other members, reflecting the low sequence similarity from the BLASTp analysis (Figure 2).

Members in this major clade of the PL7 family has not yet been classified in a subfamily in CAZy. The other known fungal PL7 sequences cluster together in a different clade that is classified as subfamily 4 in CAZy (Figure 2), which only contain glucuronan lyases (EC 4.2.2.14) [39].

**Figure 3:**
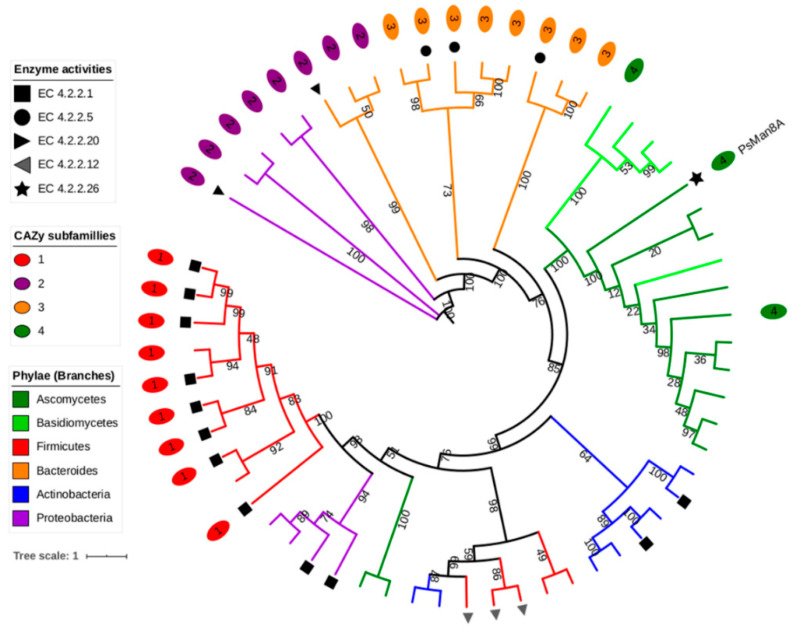
Maximum likelihood phylogenetic tree of selected PL8 protein sequences from the CAZy database including subfamily and EC number classifications. Due to the limited number of reported fungal PL8 sequences in CAZy, some additional putative fungal PL8 sequences were added from GenBank, found by Blastp analysis and subsequent PL8 HMM analysis. Enzyme activities correspond to EC numbers as follows; 4.2.2.1 = hyaluronate lyase, 4.2.2.5 = chondroitin AC lyase, 4.2.2.20 = chondroitin ABC lyase, 4.2.2.12 = xanthan lyase, 4.2.2.26 = Exo-acting polyM lyase. Numbers on branches represent bootstrap values. The tree scale bar indicates substitution changes per site. A dendrogram including accession numbers for all sequences is shown in Figure S2.

The phylogenetic analysis of the native sequence of PsMan8A clustered in a large solitary fungal clade with PL8 sequences exclusively from terrestrial fungi. The clade separation of the PL8 phylogenetic tree was strongly governed firstly by enzyme specificity and secondarily by taxonomy, this observation is corroborated by the high bootstrap values in the major clade formations. The fungal clade is unique in the respect that it contains no other functionally characterized members. PsMan8A was placed on a solitary branch in the fungal clade, which most likely signifies the lack of sequences in CAZy with similar functions, which is plausible considering PsMan8A is the only sequence originating from a marine fungus (Figure 3).

### 3.5. Expression and Purification

All recombinant enzymes were successfully expressed in *P. pastoris* and purified to 95% homogeneity, assessed by the absence of multiple bands on the SDS-PAGE gel (Supplementary material, Figure S1A). The molar weights derived from the SDS-PAGE gel tracks of PsAlg7A and PsAlg7C were in accord with their predicted sizes (approximately 25.5 kDa). However, both PsAlg7B and PsMan8A appeared to migrate less than expected, presenting a higher molar weight than anticipated. We ascribe this to be partly a result of glycosylation, which is in accordance with predicted *N*-glycosylation sites (Supplementary material Table S1), and partly slightly retarded protein mobility during the electrophoresis. After deglycosylation with EndoH, the molar weights of PsAlg7B and PsMan8A matched their predicted sizes (approx. 26 and 85.3 kDa) (Supplementary material, Figure S1B).

### 3.6. Biochemical Characterization

PsAlg7A, -B, and -C all showed activity on commercial alginate, polyM, and polyG. PsMan8A only showed significant activity on polyM. None showed activity on chondroitin AC, chondroitin B, hyaluronic acid, or polyMG under the selected conditions.

**Figure 4:**
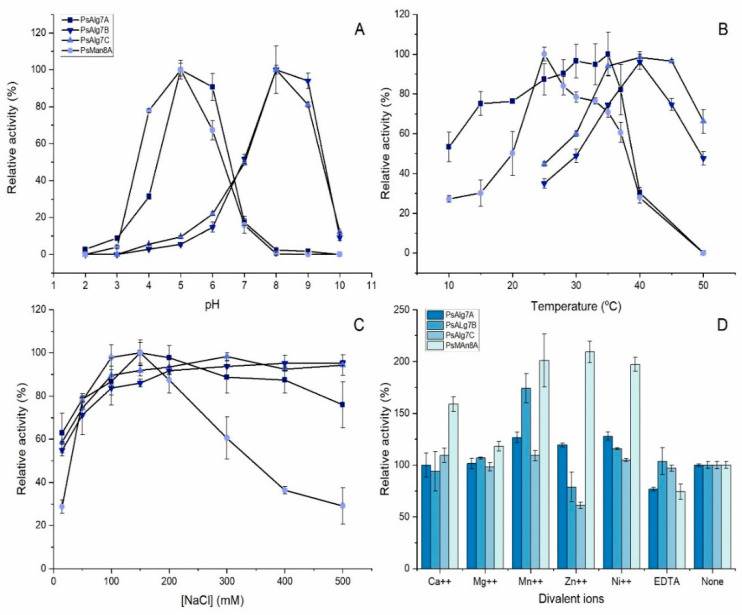
Biochemical characterization of the four alginate lyases PsAlg7A, -B, -C, and PsMan8A on polyM under std. assay conditions: (**A**) pH-optimum; (**B**) temperature-optimum; (**C**) effect of NaCl; (**D**) effect of divalent ions and EDTA with 150 mM NaCl. Error bars represent standard errors on triplicates.

The pH optimum of the alginate lyases was determined to be pH 5 for PsAlg7A and PsMan8A, and pH 8 for PsAlg7B and PsAlg7C, showing classical bell-shapes for all enzymes (Figure 4A). The temperature optimum for PsAlg7A was 35 °C, but this enzyme showed more than 80% activity in the range of 25–37 °C. PsAlg7B and PsAlg7C had distinct optima at 40 °C. PsMan8A also had a very distinct optimum temperature at 25 °C (Figure 4B).

The effect of NaCl was investigated and showed that none of the enzymes required NaCl to function, but a 40–70% increase in activity was observed in presence of NaCl. PsALg7A, -B, and -C showed the highest activity at around 200 mM, which only decreased slightly with increasing concentrations as high as 500 mM. PsMan8A showed a sharp peak at 150 mM and then a steep decrease of relative activity to 30% at 500 mM NaCl (Figure 4C).

The effects of metal ions were also investigated and surprisingly the activity of PsMan8A was promoted by all the tested metal ions (Figure 4D). Mn^++^ and Ni^++^ promoted activity of all the alginate lyases. Zn^++^ increased activity of PsAlg7A with 20% and PsMan8A with 110%, but interestingly, presence of Zn decreased the activity of PsAlg7B and PsAlg7C. EDTA decreased activity for all the enzymes (Figure 4D).

### 3.7. Kinetic Parameters of PsAlg7A, PsAlg7B, PsAlg7C, and PsMan8A

The plots of initial rate kinetics on increasing substrate concentrations showed classical Michaelis–Menten curves for PsAlg7A, -B, and -C on the substrates alginate and polyM. The same applied for PsAlg7B and -C on polyG, however, for PsAlg7A the plot appeared linear (Figure S3). The lack of saturation could suggest that the observed activity may have been due to residual polyM chains in the substrate. Indeed, when adjusting the substrate concentrations to 18% of the measured amounts, and comparing the initial velocities of the adjusted polyG to the ones observed on polyM the slopes of *v*_0_ were quite similar. For these reasons we chose to omit the apparent kinetic parameters for PsAlg7A on polyG in Table 1. For all three recombinant PL7 enzymes the lower substrate affinity was reflected in the 5–10 fold higher *K*_m_ values and the correspondingly lower specificity constants (*k*_cat_/*K*_m_) on polyG, when compared to alginate and polyM (Table 1). The highest catalytic efficiency for all enzymes was found on polyM, followed closely by alginate for the PL7 alginate lyases. This suggests that M-blocks are the preferred substrate for all the enzymes. Interestingly, PsMan8A showed significant activity exclusively on polyM, strongly indicating this enzyme to be strictly exo-acting and therefore finding only few to none accessible M-block attack-sites on alginate. The specific activity of PsMan8A was the lowest of all despite this enzyme having the second highest *k*_cat_. The explanation of course being that the specific activity does not take the size of the enzyme into account in which case the specific activity of PsMan8A would be approximately three times higher.

Due to differences in type of assay used in different reports, definitions of units and the general lack of kinetic parameters for alginate lyases in the literature, major comparisons of catalytic rates and specific activities of alginate lyases are difficult. However, it seems the *P. salina* alginate lyases compare similarly to other PL7 alginate lyases. The reported specific activities of several of members of the clade shared with PsAlg7A, -B, and -C, range between 1400 and 1600 U/mg. This includes the PL7 from the red algae *Pyropia yezoensis* [17] and two bacterial ones from a *Vibrio* sp. [40] and a *Nitratiruptor* sp. [41]. The endolytic PL7 enzyme AlyA1 from the marine bacterium *Zobellia galactanivorans* had a *k*_cat_ between 12 and 19 s^−^^1^ (dependent on the polyG concentration in alginate), K_m_ value between 1.7 and 6 mM, and catalytic specificity constant (*k*_cat_/*K*_m_) between 3 and 7. 

### 3.8. Synergy of PsAlg7A and PsMan8A

The specificity of PsMan8A towards polyM, prompted us to investigate any possible endo-exo synergy effect on alginate between PsAlg7A and PsMan8A as they share pH optima, immediate genetic location, and was found to be co-secreted by *P. salina* when grown on several different brown algae species under similar conditions [22].

**Figure 5:**
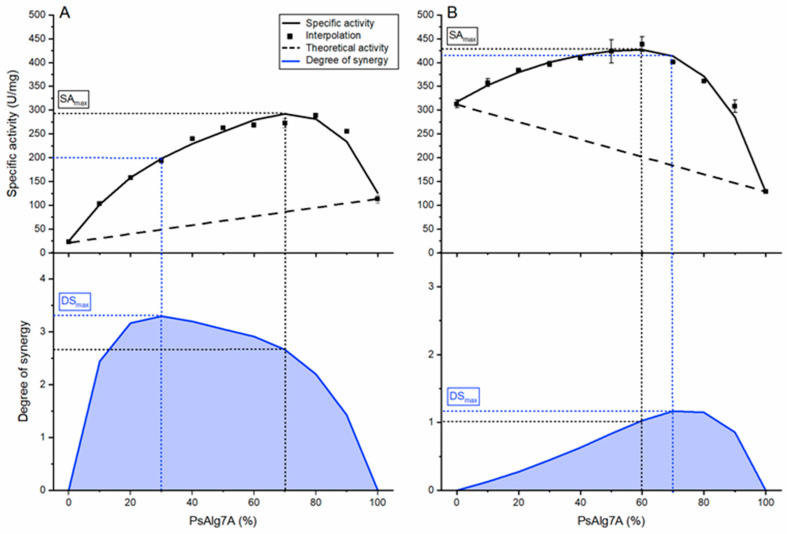
Endo-exo synergy experiment of PsAlg7A and PsMan8A. The total enzyme loads were at all points 0.2 μM with varying ratios the two enzymes indicated by the % PsAlg7A on the x-axis which indicates the load of PsMan8A indirectly (% PsMan8A = 100%—% of PsAlg7A). The dashed line on the top panels represents the theoretical specific activities (SA) without the effect of synergy. The solid line through the specific activity data points in the top panels represents spline fits, which were used with the theoretical specific activity for calculating the curve in the bottom panels showing the degrees of synergy (DS). DS_max_ = maximum degree of synergy. SA_max_ = maximum specific activity. Error bars represent standard deviation from triplicate experiments: (**A**) Alginate (1.5 mg·mL^−1^); (**B**) PolyM (1.5 mg·mL^−1^).

The degree of synergy (DS) was defined as the ratio between the measured specific activity (SA) and the theoretical, linear specific activity increase in response to an additive effect of enzyme load [38]. By that definition the DS_max_ is thus the optimal ratio between two enzymes at the chosen enzyme load where the enzymes cooperate the most effectively and obtain the highest difference between the measured specific activity of the two enzymes and their theoretical (additive) specific activity. The DS_max_ and optimal ratios may change with increasing substrate concentrations, enzyme concentrations, and assay time as observed with cellulases [38]. The objective with this experiment was however not to perform an exhaustive hunt for the optimal conditions of synergy, but to introduce the phenomena and illuminate the relationship and roles of PsAlg7A and PsMan8A further. On alginate the DS_max_ reached 3.2 in this setup with the ratio of 30% PsAlg7A and 70% PsMan8A (Figure 5A). The negative slope of the theoretical specific activity on polyM (Figure 5B) is an effect of the higher turnover rate of PsMan8A compared to PsAlg7A since the obstacle of alginate recalcitrance no longer applies. There is however still a slight synergy effect seen, probably on account of the lack of substrate saturation for PsMAn8A thereby allowing PsAlg7A to provide some additional attack sites on polyM. As observed with cellulases the DS_max_ and SA_max_ is not found at the same ratio of the two enzymes [38]. SA_max_ is governed by the turnover rate of the most effective enzyme. This does not necessarily mean that this is the point where the two enzymes work together most optimally. On alginate PsMan8A is entirely dependent on PsAlg7A to deliver attack-sites and therefore it is the least effective enzyme on alginate resulting in a low theoretical specific activity when approaching 100% PsMan8A.

An obvious interpretation of the synergy results (Figure 5) is that the endo-acting PsAlg7A provides attack sites to the exo-acting PsMan8A and together the enzymes thus catalyze degradation of the substrate further and at a higher rate than when each enzyme is acting on their own. On alginate the exo-acting PsMan8A has almost no activity because of the lack of available polyM chain ends until the endo-acting PsAlg7A provides fresh attack sites. On pure polyM the DS most likely represents the distance in concentration to the substrate saturation point of PsMan8A. The significant synergy (as opposed to just an addition effect) indicates that the PsMan8A indeed benefits from the apparent polyM preference of PsAlg7A (Table 1), and implies that PsMan8A may attack the nonreducing ends containing terminal unsaturated polyM. However, the synergy may also include a true “kinetic effect” as shorter oligosaccharides move faster and may impart lesser microenvironmental viscosity effects than longer chains.

### 3.9. Product Profiles of P. salina Alginate Lyases

**Figure 6:**
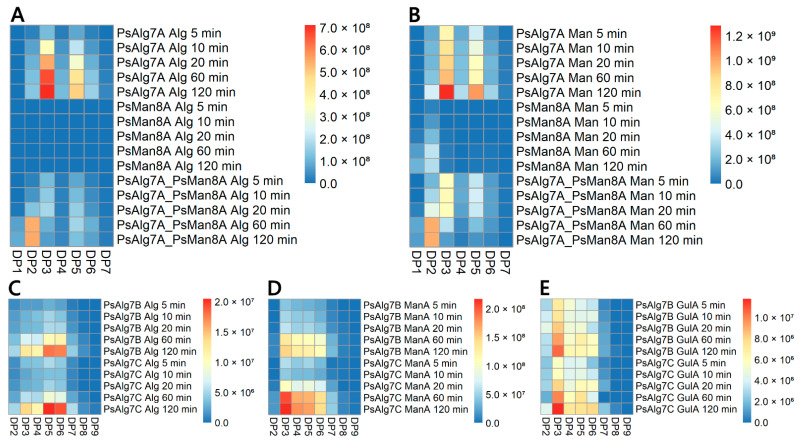
Heat maps of averaged relative intensities derived from the LC-ESI-MS analysis of time-course experiments for the alginate lyases: (**A**) PsAlg7A and PsMan8A acting on alginate alone and in synergy; (**B**) PsAlg7A and PsMan8A on polyM alone and in synergy; (**C**) PsAlg7B and PsAlg7C on alginate; (**D**) PsAlg7B and PsAlg7C on polyM; (**E**) PsAlg7B and PsAlg7C on polyG. Barchart representations of the data including the standard errors from triplicate experiments can be found in the Supplementary Materials, Figure S4. Example chromatograms from PsAlg7A and PsMan8A on polyM with thorough descriptions can be found in the Supplementary Materials, Figures S5–S8.

The product profile of PsAlg7A on alginate and polyM (Figure 6A,B) showed an accumulation over time of products ranging from 7 to 2 degree of polymerization (DP) with a clear accumulation of DP5 and DP3 oligomers consistent with endo-action (see also Figures S4 and S5). As seen in the substrate screening, PsMan8A showed no detectable product formation on alginate, but on polyM, DP2 and DP1 were formed exclusively (Figure 6A,B, Figures S4-I and S6). In general, unsaturated monosaccharides like DP1, and its nonenzymatic conversion product 4-deoxy-L-erythro-5-hexoseulose urinate (DEH), are poorly ionized, which results in decreased intensity especially at the target mass of 500 Da in the smart parameter setting applied in this study. Hence, the accumulation of DP1 intensities were lower than DP2. The formed products in the synergy experiment between PsAlg7A and PsMan8A on both alginate and polyM (Figure 6A,B) showed an accumulation of DP3 products the first 20 min matching the product profile of PsAlg7A, followed by a decrease as those oligomers became depolymerized to DP2 and DP1 by PsMan8A. These results signify that PsMan8A effectively depolymerizes polyM chains of varied lengths, from long chains down to DP3, creating only DP2 and DP1 sized products, indicative of exo-action. In contrast, PsAlg7A, B, and C all catalyze depolymerization of alginate, polyM, and polyG to oligomers, without monomer formation, indicative of endo-action.

PsAlg7B and PsAlgC showed very similar product profiles. Interestingly DP6 and DP5 oligomers were the main products on alginate (Figure 6C), but on polyM and polyG, DP3 oligomers were prominent (Figure 6D,E). This may speak to the previously mentioned recalcitrance of alginate versus the more easily accessible purified constituents. As with PsAlg7A, B and C also produced a minor fraction of DP2 products, but significantly more on polyG than on polyM and alginate (Figure 6C–E).

## 4. Discussion

Given the abundance of brown algae in the marine environment and high amount of alginate in their cell wall [42], it is surprising that alginate degradation by marine fungi is a relatively rarely observed phenomenon [8,43]. Part of the explanation could very well be that marine fungi are insufficiently studied, lacking sequenced genomes and recombinant protein studies. Indeed, only very few detailed characterizations of fungal alginate lyases exist, none of which provides the sequence information [44,45] making it difficult to replicate or to build upon. The presence of three PL7 sequences in the *P. salina* genome is by itself not surprising given the wealth of putative fungal PL7 sequences found in GenBank. However, the phylogenetic relationship of these three sequences in a clade dominated almost solely by bacterial alginate lyases raises interesting questions about the origin of these genes. Whether they are the result of an ancient horizontal transfer event from unknown marine bacteria, as observed for the brown algae *Ectocarpus siliculosus* [42], or possibly the result of convergent evolution on the basis of the same PL7 ancestor as the terrestrial fungi remains an open question. Both scenarios support the hypothesis that *P. salina* evolved from a terrestrial fungus. Indeed the *P. salina* genome harbors a large amount of transposable elements compared to its close terrestrial relatives, suggesting that it has a very high ability for rearranging and mutating its genes, creating new substrate specificities and discarding genes that does not serve a function in its current ecological niche [22].

Previously *P. salina’s* ability to grow with polyM as the sole carbon source has been demonstrated [46], and the discovery of PsMan8A represents genetic evidence of this ability, being the final crucial step of complete degradation of polyM to monomers necessary for efficient uptake and thus metabolization of mannuronic acid. The role of the PsMan8A and the phylogenetic placement of this sequence amongst terrestrial fungal sequences with unknown substrate specificities indicate a specific adaption for the complete degradation of polyM. The PL8 family is almost exclusively composed of bacterial endo-, exo-enzymes acting on glycosaminoglycans (GAGs), a family of linear, complex, and highly sulfated polysaccharides found in animal tissue and often observed as proteoglycans [47]. At the time of writing (December 2020) there were 2733 bacterial and nine fungal PL8 sequences (including PsMan8A) in the CAZy database. None of the fungal sequences have been characterized, suggesting that these sequences are rare or simply underinvestigated. In the phylogenetic analysis almost all the fungal enzymes formed a very distinct clade suggesting unique sequence features compared to the bacterial sequences. That the fungal enzymes form a distinct clade is corroborated by the CAZy PL8 subfamily 4 classification of members of the fungal clade, including PsMan8A. Whether some of these fungal enzymes are specific for alginate is possible, but unlikely, since all the sequences originate from fungi found in terrestrial environments, where alginate is very scarce, produced only by a few specialized bacterial genera [48]. This further underlines the uniqueness of PsMan8A and *P. salina*.

The pairwise differences in pH optima of PsAlg7A-PsMan8A (pH 5) and PsAlg7B-PsAlg7C (pH 8), and the differences in secretion levels from *P. salina* observed in the proteomic study on brown algae after 14 days of fermentation, where the amount of PsAlg7B and PsAlg7C were significantly lower than two others [22], allow for the likely hypothesis that PsAlg7B and PsAlg7C are secreted in the initial stages of fermentation, when the pH is governed by the seawater, which matches their optima. As the fungus’ hyphae dig further into the alginate rich exocellular layers of the brown algae, the micro-environment changes. In turn, pH is lowered, perhaps by the release of products from PsAlg7B and PsAlg7C or by the production of acids from the fungus, PsAlg7A and PsMan8A are secreted and act in effective synergy to completely depolymerize the polyM portion of the now disrupted alginate, thereby granting the fungus a carbon source and exposing the other polysaccharides of the brown algae cell wall matrix. This hypothesis fits well with the effect of NaCl on PsAlg7B and PsAlg7C, which showed no significant decline in activity as the salinity in the reactions approached that of seawater (600 mM) [49]. The potential interest of the fungus for an initial fast and effective disruption of alginate is mirrored in the high specificity constants (*k*_cat_/*K*_m_) of PsAlg7B and PsAlg7C, which were more than an order of magnitude higher on alginate and polyM compared to those on polyG, yet the *k*_cat_ values of PsAlg7C on alginate, polyM, and polyG were similar. A recent study using immune-localization revealed the that the alginate showed layered properties with the polyG-rich layer as the outermost followed by a MG-rich and finally a polyM-rich layer [11], which to some extent matches the substrate specificities of the four alginate lyases from *P. salina*. The product profile of PsAlg7B and PsAlg7C revealed the depolymerization of polyG to produce dimers, which may be transported into the cell by an unknown MFS transporter, where it can act as a signaling molecule to initiate expression of more alginate lyases, as observed with other sugar dimers in terrestrial fungi [50].

## 5. Conclusions

In the present study we expressed and purified four novel fungal alginate lyases from *P. salina* and characterized them in depth. Our findings reveal the action and substrate specificity of the *P. salina* PL8 lyase (PsMan8A) and show with a high degree of certainty that the mode of action of this enzyme is exo-action on polyM. A significant synergistic effect between the PsMan8A and the endo-acting PsAlg7A was also observed, corroborating the suggested exo-action of the PsMan8A. To our knowledge this is the first report describing the characterization of several new sequence-derived recombinant fungal alginate lyases and notably a first discovery of an exo-polyM acting PL8 alginate lyase.

This study provides new and detailed insights about fungal derived alginate lyases. The data provide a new foundation for understanding how marine fungi accomplish depolymerization of alginate to support growth on brown macroalgae, and may furthermore inspire development of new biotechnology applications of *P. salina* alginate lyases for tailoring alginate oligomers for novel uses.

## Figures and Tables

**Table 1:** Kinetic parameters and specific activities (SA) for the four alginate lyases on commercial substrates. The parameters were calculated by fitting the Michaelis–Menten model in the program Origin. ± represents standard errors on triplicates.

Enzyme	Parameters	Alginate	PolyM	PolyG
PsAlg7A	SA (U mg^−1^)	245 ± 4.4	1263 ± 6.5	n/a
	*K*_m_ (mM)	3.9 ± 0.1	17.4 ± 0.3	n/a
	*k*_cat_ (s^−1^)	0.65 ± 0.01	3.4 ± 0.02	n/a
	*k*_cat_/*K*_m_	0.16	0.19	n/a
PsAlg7B	SA (U mg^−1^)	1008 ± 21	1459 ± 6.8	832 ± 22
	*K*_m_ (mM)	1.3 ± 0.1	1.7 ± 0.1	5.0 ± 0.1
	*k*_cat_ (s^−1^)	2.6 ± 0.05	3.8 ± 0.02	2.1 ± 0.06
	*k*_cat_/K_m_	2.07	2.16	0.43
PsAlg7C	SA (U mg^−1^)	2889 ± 31.1	4481 ± 47	3267 ± 39
	*K*_m_ (mM)	1.8 ± 0.1	2.4 ± 0.1	22.0 ± 0.1
	*k*_cat_ (s^−1^)	7.3 ± 0.079	11 ± 0.12	8.3 ± 0.10
	*k*_cat_/K_m_	4.1	4.7	0.4
PsMan8A	SA (U mg^−1^)	nd.	1093 ± 17	nd.
	*K*_m_ (mM)	nd.	21.0 ± 0.7	nd.
	*k*_cat_ (s^−1^)	nd.	9.7 ± 0.2	nd.
	*k*_cat_/K_m_	nd.	0.5	nd.

## Supplementary Materials

The following are available online at https://www.mdpi.com/2309-608X/7/2/80/s1, Table S1: Protein characteristics, Figure S1: SDS-PAGE of the purified recombinant enzymes, Figure S2: Dendrogram rendering of the maximum likelihood phylogenetic tree of PL8 sequences, Figure S3: Dot plots of initial velocities of the recombinant enzymes, Figure S4-I-III: Barcharts of the averaged intensities from the LC-ESI-MS analysis, Figure S5: Elution profile of observed structures from PsAlg7A acting on polyM after 120 min., Figure S6: Extracted ion chromatogram and fragmentation patterns from PsMan8A acting on polyM after 120 min., Figure S7. Ion chromatograms from PsAlg7A acting on polyM, Figure S8: Ion chromatograms from PsMan8A acting on polyM.
