# Supplementary material for: Specificities and Synergistic Actions of Novel PL8 and PL7 Alginate Lyases from the Marine Fungus Paradendryphiella salina
Source: J Fungi (Basel). 2021 Jan 25;7(2):80. doi: 10.3390/jof7020080 (PMC7911691; doi:10.3390/jof7020080)
Supplement: Supplementary file 1 [file jof-07-00080-s001.pdf]

Supplementary file 1

Table S1. Protein characteristics based on the recombinant sequences calculated by ProtParam and NetNGlyc.

Protein-name	Amino acids	N-glycosylation sites	Molar mass kDa	Theoretical pI	Ext. coefficient (ε [$M^{-1}cm^{-1}$])
PsAlg7A	231	1	25.446	4.46	30035
PsAlg7B	233	1	25.995	5.20	36900
PsAlg7C	234	1	25.852	4.86	36565
PsMan8A	773	10	85.307	4.48	151190

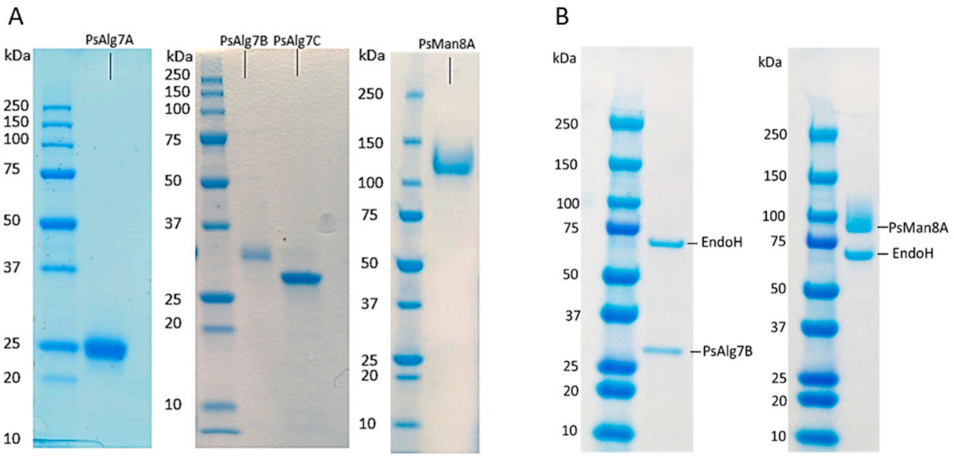

Figure S1. SDS-PAGE of the purified recombinant enzymes: (A) Untreated purified enzymes; (B) PsAlg7B and PsMan8A after EndoH deglycosylation treatment.

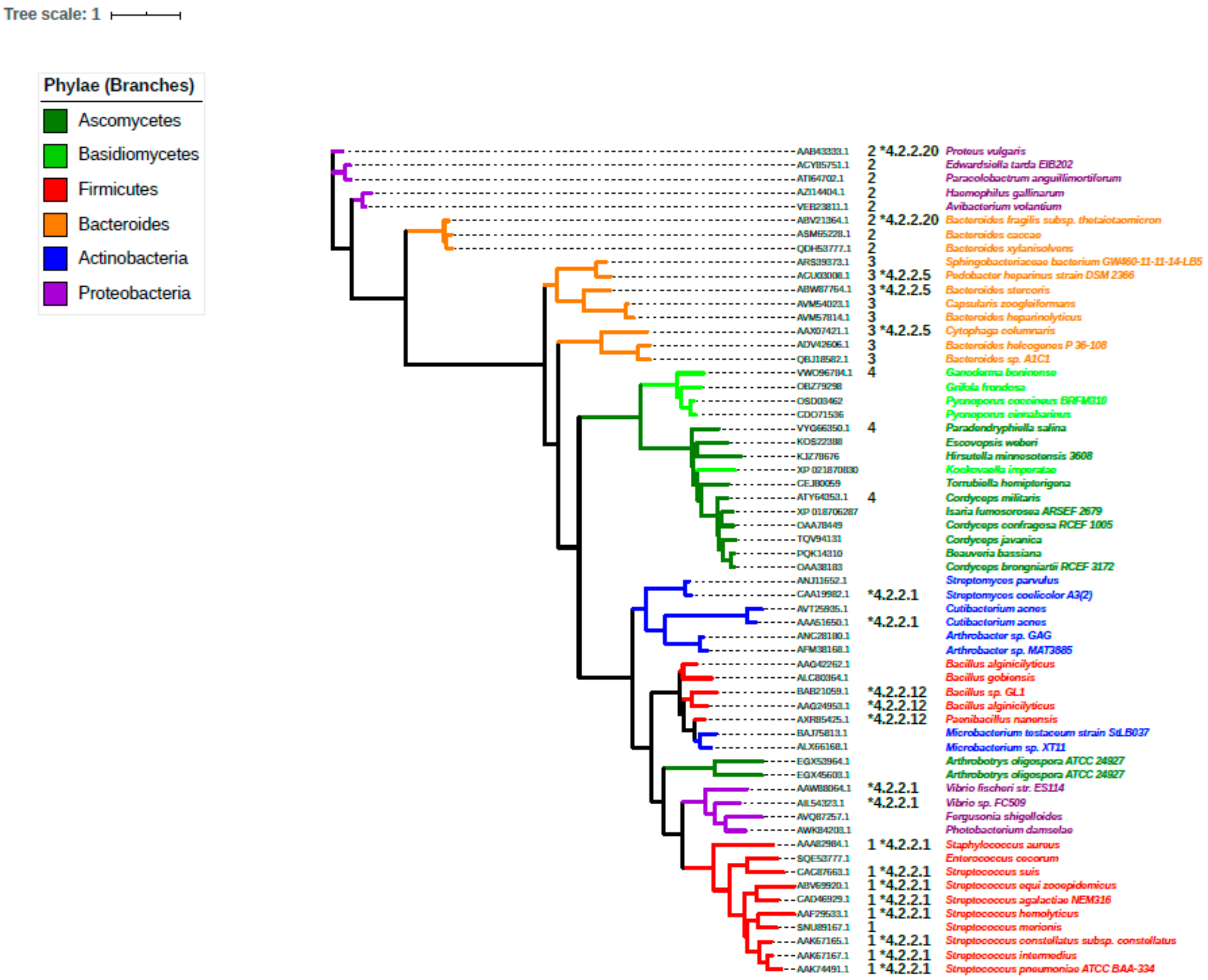

Figure S2. Dendrogram rendering of the maximum likelihood phylogenetic tree of PL8 sequences. Genbank accession numbers are placed at the ends of the nodes.

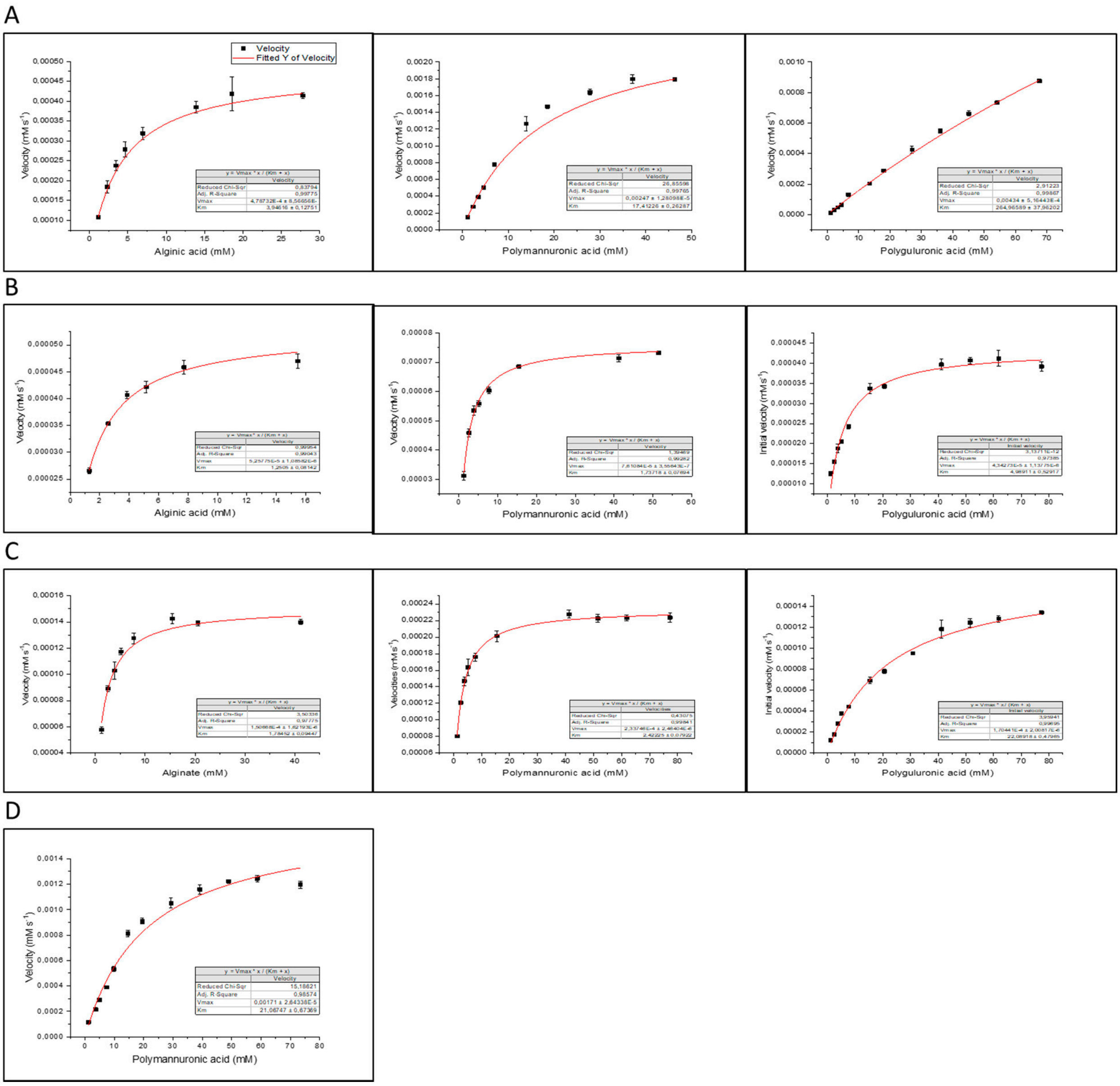

Figure S3. Dot plots of initial velocities vs. substrate concentration with the Michaelis-Menten model fitted by Origin (red line): (A) PsAlg7A; (B) PsAlg7B; (C) PsAlg7C; (D) PsMan8A.

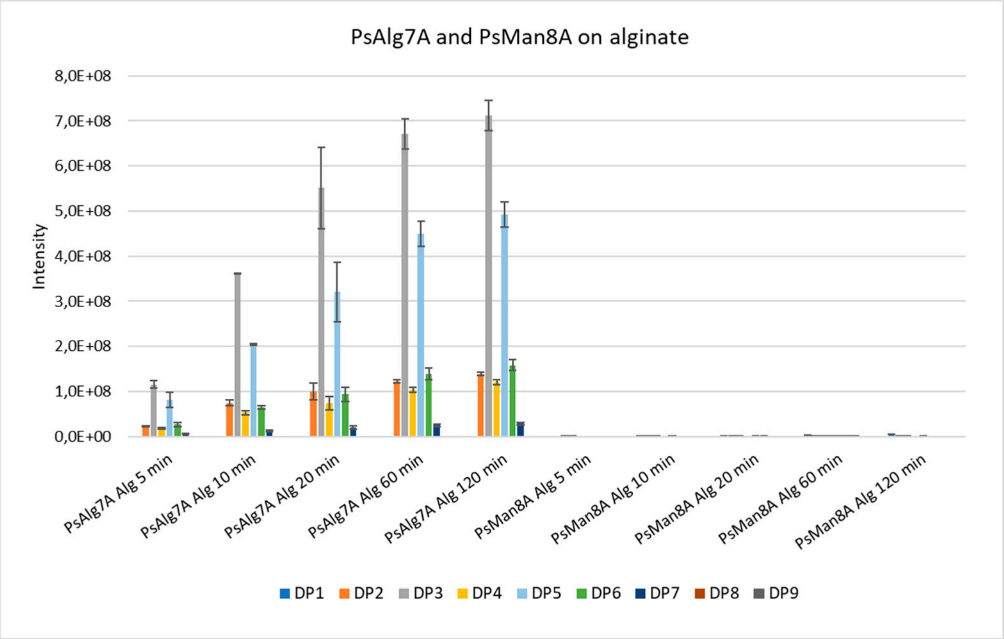

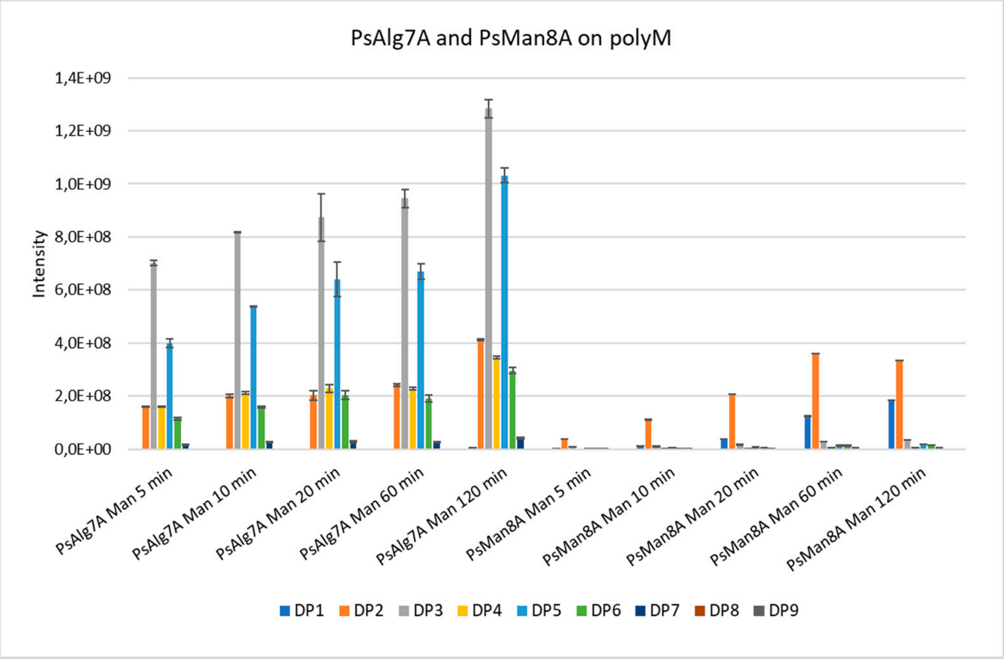

Figure S4-I. Barcharts of the averaged intensities from the LC-ESI-MS analysis of time-course experiments for the alginate lyases. These data show the underlying values represented in the Figure 6 heat maps in the main article: Here, specifically the results for the action of the individual enzymes PsAlg7A and PsMan8A on alginate and polyM, respectively (explanations behind the LC-ESI-MS data interpretations are outlined in Figures S5-S8, below). Error bars represent standard deviation from triplicate experiments.

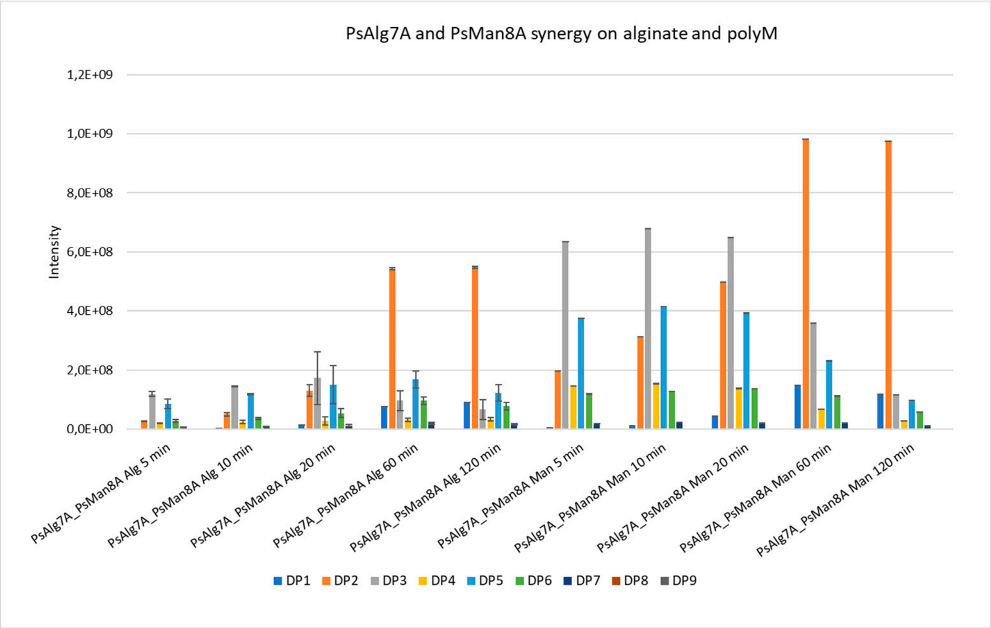

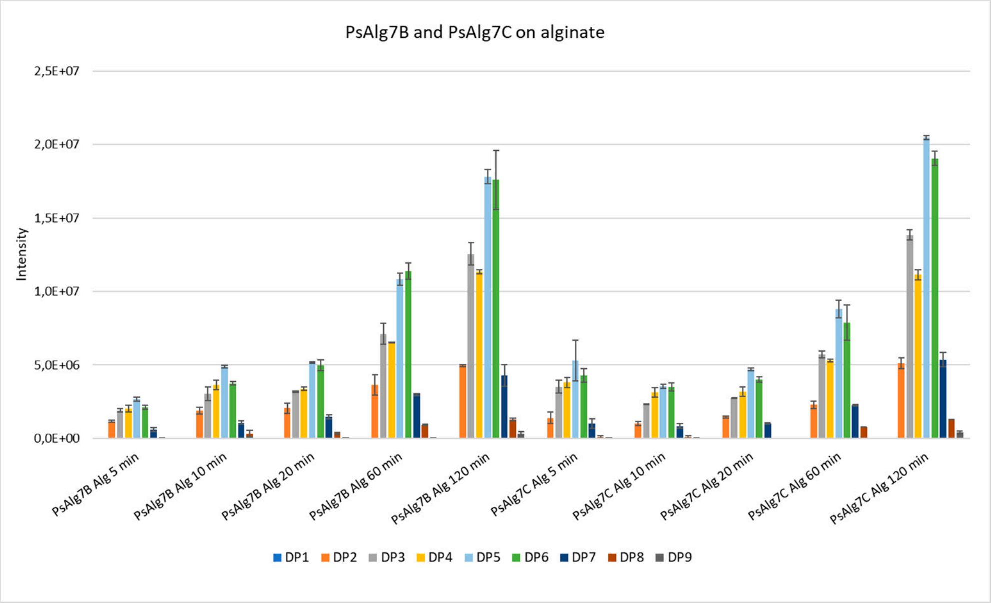

Figure S4-II. Barcharts of the averaged intensities from the LC-ESI-MS analysis of time-course experiments for the alginate lyases. These data show the underlying values represented in the Figure 6 heat maps in the main article: Here, specifically the results for the combinations of enzymes PsAlg7A and PsMan8A on alginate and polyM, respectively (upper panel) and the data for the individual actions of PsAlg7B and PsAlg7C on alginate (lower panel). Error bars represent standard deviation from triplicate experiments.

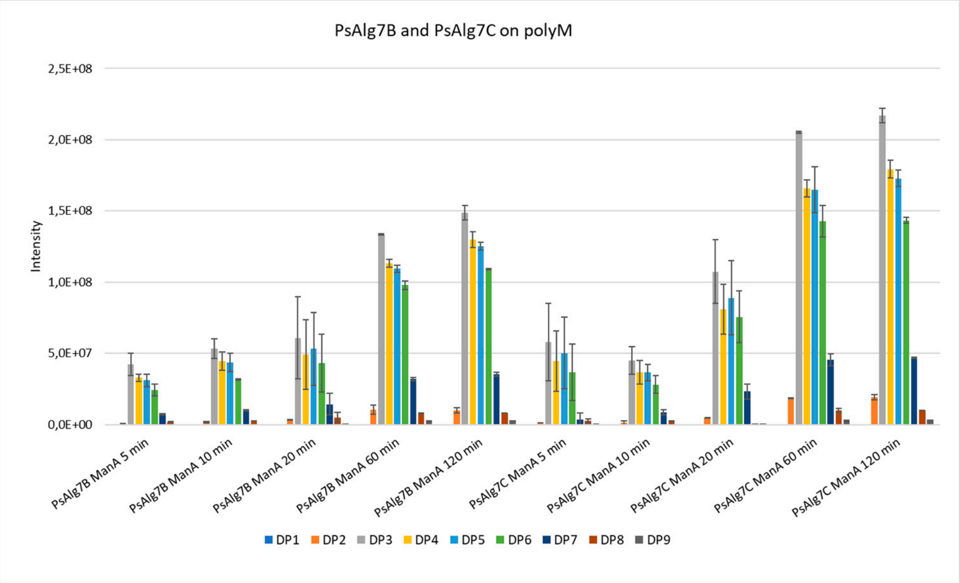

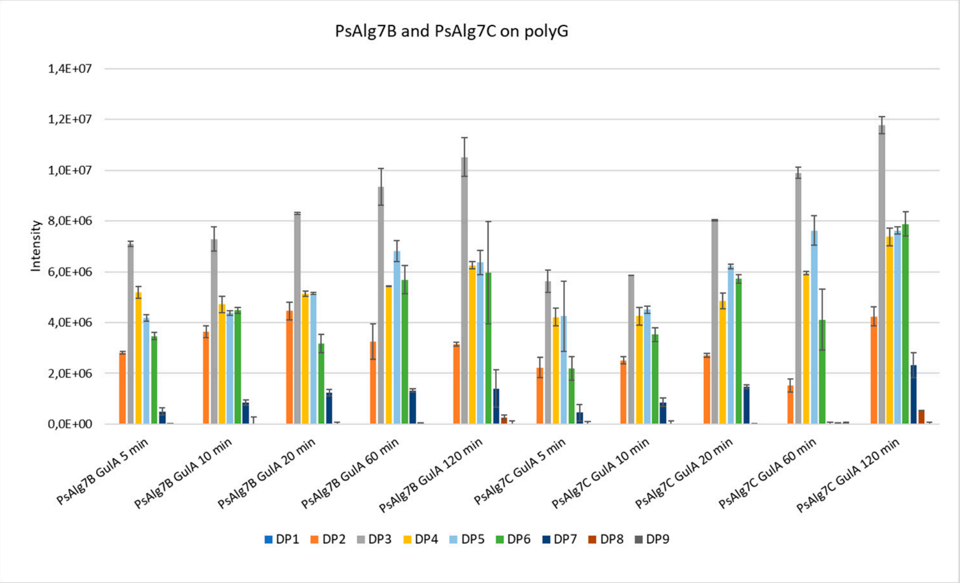

Figure S4-III. Barcharts of the averaged intensities from the LC-ESI-MS analysis of time-course experiments for the alginate lyases. These data show the underlying values represented in the Figure 6 heat maps in the main article: Here, specifically the results for the enzymes PsAlg7B and PsAlg7C acting on polyM (upper panel) and on polyG (lower panel). Error bars represent standard deviation from triplicate experiments.

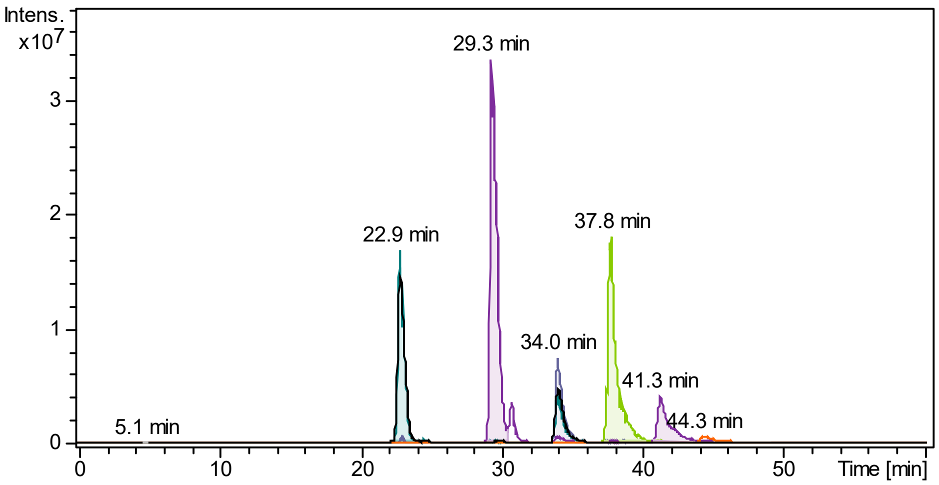

Figure S5. Elution profile of observed structures from PsAlg7A acting on polyM after 120 min. Extracted ion chromatograms of DP1-7 corresponding to retention times of DP1: 5.1 min, DP2: 22.9 min, DP3: 29.3 min, DP4: 34.0 min, DP5: 37.8 min, DP6: 41.3 min, DP7: 44.3 min.

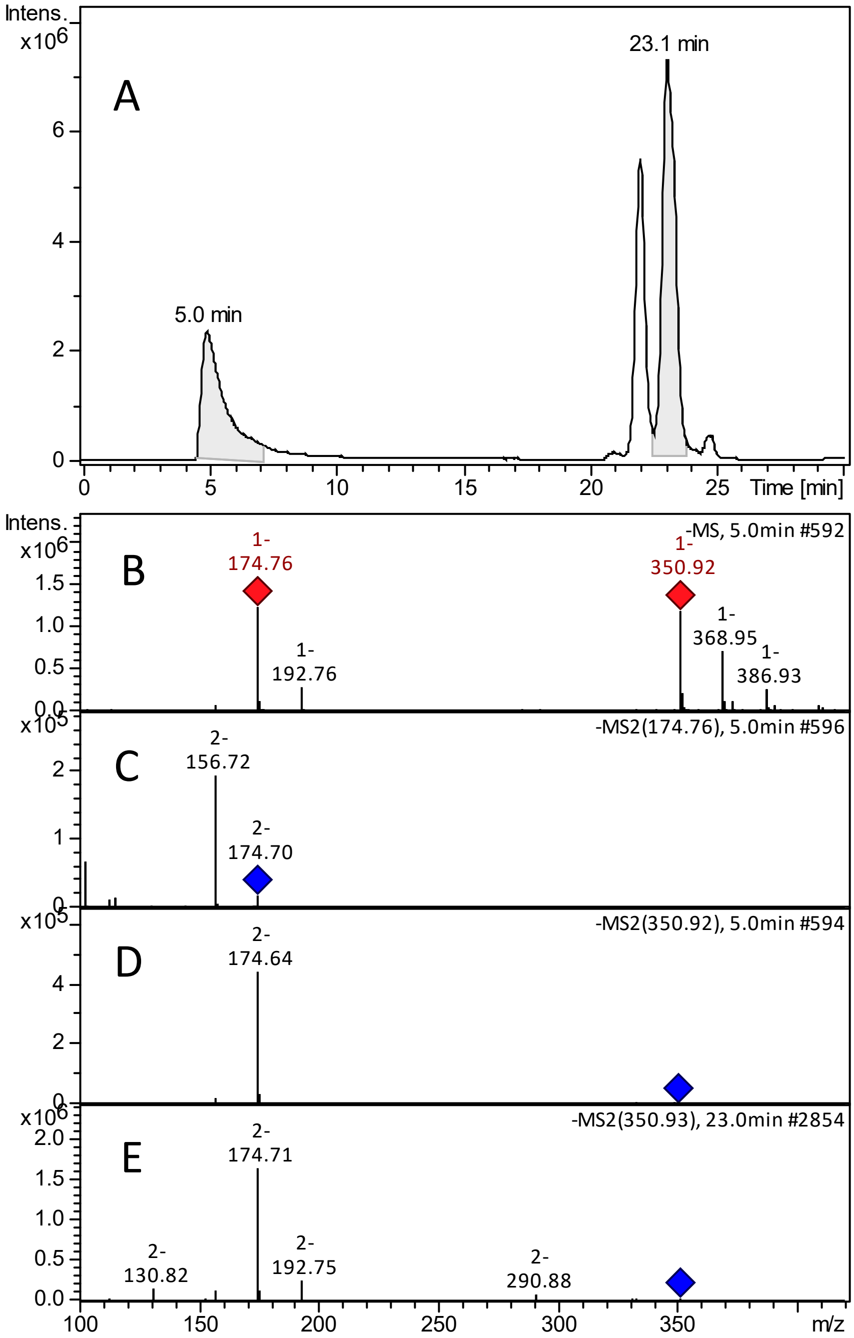

Figure S6. A: Extracted ion chromatogram of 176 and 351 m/z from PsMan8A acting on polyM after 120 min indicating DP1 at 5.0 min, DP2 at 23.1 min (0.1-0.2 min shift in retention time, compared to figure S5) B: full scan at 5.0 min. Red rhombus indicates the most abundant and second most abundant peaks. C: Fragmentation pattern of 175 m/z at 5.0 min. Blue rhombus indicates mother ion. D: Fragmentation pattern of 351 m/z at 5.0 min. Blue rhombus indicates mother ion. E: Fragmentation pattern of 351 m/z at 23.0 min. Blue rhombus indicates mother ion.

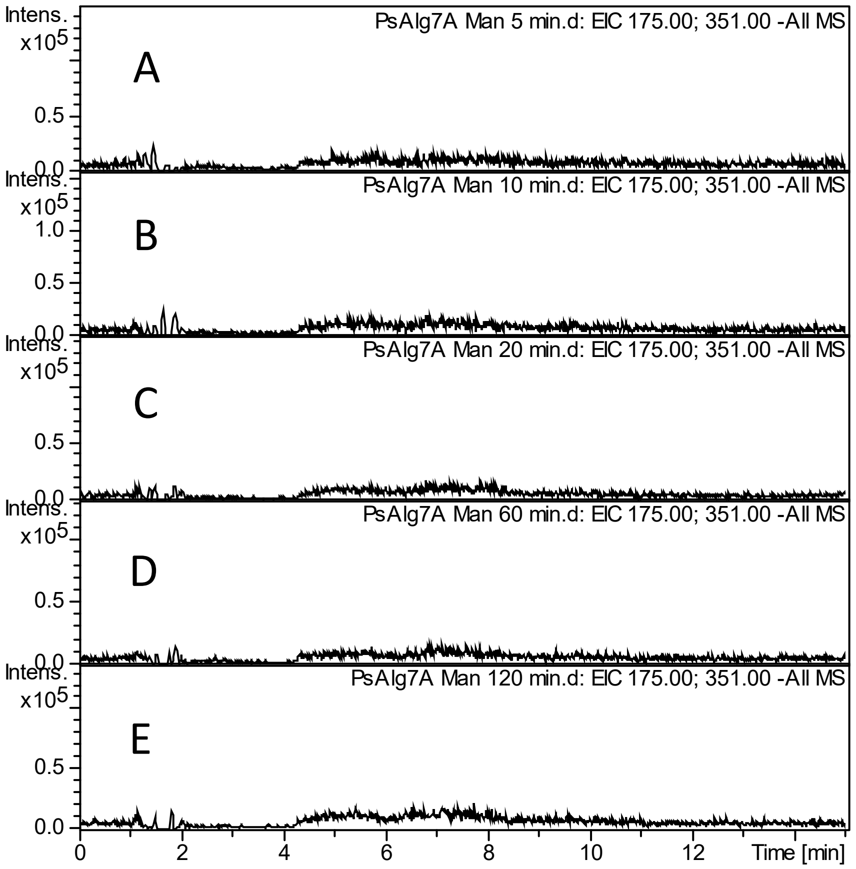

Figure S7. Product development of DP1 over time. Ion extracted chromatograms of 175 and 351 m/z in reactions of PsAlg7A acting on polyM after A: 5 min, B: 10 min, C: 20 min, D: 60 min, E: 120 min. The time course reveals no development of DP1 over time for the endo-lytic PsAlg7A.

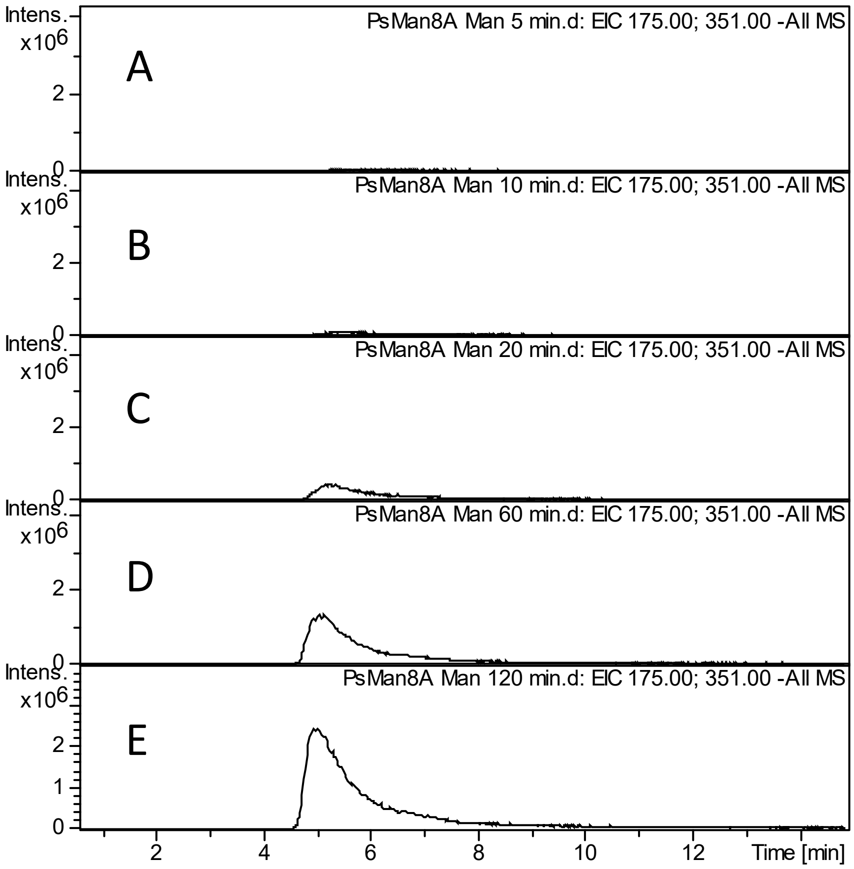

Figure S8. Product development of DP1 over time: Ion extracted chromatograms of 175 and 351 m/z in reactions of PMan8A acting on polyM after A: 5 min, B: 10 min, C: 20 min, D: 60 min, E: 120 min. The time course reveals a clear development of DP1 for the exo-lytic PsMan8A.

Notes to Supplementary Figures S5-S8

The major masses at 5.0 minutes are 175 and 351 m/z. 175 m/z correspond to the $[M-H]^{-1}$ adduct of monomeric dehydrated hexuronic acid. 351 m/z corresponds to a hexuronic acid monomer in a di-adduct state $[2M-H]^{-1}$ due to the ionization process diagnosed by the lack of fragmentation in the range between 175 and 351 m/z. Contrary to this is the fragmentation of 351 m/z at 23.0 min (Figure S6, D) showing fragments in the range between 175 and 351 m/z, indicating the presence of the $[M-H]^{-1}$ adduct of DP2. Hence, in order to quantify the formation of DP1 relatively, the extracted ion chromatogram of DP1 is comprised of both 175 and 351 m/z.
